# Organ-Specific Gene Expression Reveals the Role of the *Cymbidium ensifolium-miR*396*/Growth-Regulating Factors* Module in Flower Development of the Orchid Plant *Cymbidium ensifolium*

**DOI:** 10.3389/fpls.2021.799778

**Published:** 2022-01-27

**Authors:** Fengxi Yang, Chuqiao Lu, Yonglu Wei, Jieqiu Wu, Rui Ren, Jie Gao, Sagheer Ahmad, Jianpeng Jin, Yechun Xv, Gang Liang, Genfa Zhu

**Affiliations:** ^1^Guangdong Key Laboratory of Ornamental Plant Germplasm Innovation and Utilization, Environmental Horticulture Research Institute, Guangdong Academy of Agricultural Sciences, Guangzhou, China; ^2^CAS Key Laboratory of Tropical Plant Resources and Sustainable Use, Xishuangbanna Tropical Botanical Garden, Kunming, China

**Keywords:** orchid, *Cymbidium ensifolium*, floral organ, growth-regulating factor, MiR396

## Abstract

Orchids are some of the most popular ornamental plants worldwide. Orchid floral morphology has increasingly attracted horticultural and commercial attention. Although multiple genes have been shown to be involved in the formation of the orchid flower, the underlying multi-level regulatory networks are largely unknown. In this study, we analyzed the ontogeny of flower development in *Cymbidium ensifolium*, a traditional orchid in the tropical and subtropical regions of Asia, by performing deep sequencing of the transcriptome of individual flower organs to discover organ-specific genes potentially involved in their growth. We identified 3,017 differentially-expressed genes (DEGs) during the development of various flower organs, and observed over-representation of *GROWTH-REGULATING FACTORS* (*GRFs*) specific to flower column (gynostemium). Eleven *C. ensifolium GRFs* (*CeGRFs*) from our transcriptome data clustered into five phylogenetic subgroups. Ten of these *GRFs* shared a region complementary to *C. ensifolium* microRNA396 (*Ce*-miR396), and degradome sequencing confirmed the cleavage of transcripts derived from seven *CeGRFs*. We cloned *Ce-*miR396 and used a protoplast-based transient expression system to overexpress it in *Cymbidium* protoplasts. We observed a significant decrease in the transcripts of several *CeGRFs* in flowers and leaves, indicating a potential role for miR396–GRF module in organ development through the cleavage of distinct *CeGRFs*. Temporal and spatial expression analysis indicated that most *CeGRF* transcripts accumulated in flower buds and column tissues, where *Ce*-miR396 expression was the lowest. Expression dynamics in wild type and floral-defective mutants further confirmed a strong correlation between *Ce*-miR396, *CeGRFs*, and flower organ development and column specification. Moreover, overexpression of *Ce*-miR396 in *Nicotiana tabacum* resulted in curved pistils and reduced fertility, implying that the conserved role of *Ce*-miR396 in floral development. These results provide tools to better understand the biological roles of *GRFs* in orchid development, and open new avenues for the diversification of orchid floral patterns.

## Introduction

Orchids are a highly valuable floricultural crop. Comprising more than 25,000 species distributed in ∼800 genera, orchids represent one of the largest families of flowering plants (Ramirez et al., 2007; Givnish et al., 2015). Over the past 1,500 years, more than 70,000 orchid hybrids and cultivars have been grown as ornamentals and medicinal plants, as well as food flavoring agents and essential oils (Wang et al., 2015; Li et al., 2017; Su et al., 2018). In China, Japan, South Korea, and Southeast Asia, orchids of genus *Cymbidium* are prized for their beautiful, fragrant flowers and elegant leaves. In particular, *Cymbidium ensifolium*, which belongs to subgenus Jensoa, blossoms many times a year and is thus highly valuable in flower markets of China (Zhang et al., 2015; Yang et al., 2017; Su et al., 2018).

Orchid flower is an important ornamental and industrial material. A standard orchid flower is composed of three petal-like sepals in the first whorl, two lateral petals, a specialized bottom petal (called lip or labellum) in the second whorl, and a column (also called gynostemium) formed by the fusion of pistil, stigma and pollinia in the middle of the flower. Homeotic mutations occur frequently in orchid family, that diversify their spectacular floral morphology (Aceto and Gaudio, 2011; Yang et al., 2017; Xiang et al., 2018). The molecular underpinnings of flower development in terms of floral organ number, arrangement, and initiation timing has been widely studied in the orchid family. A unique mechanism underlying perianth patterning of orchid plants is mediated by MADS-box type transcription factors (TFs) with some modifications of the arabidopsis ABCE model of flower development (Hsu et al., 2015). In addition, many floral meristem identity genes have been functionally characterized, including *FLOWERING LOCUS T* homologs (*Phalaenopsis aphrodite, Oncidium* Gower Ramsey, *Dendrobium nobile*), *CONSTANS-like genes*, *LEAFY* (*Phalaenopsis aphrodite)*, and coregulated transcription factors such as *CINCINNATA-like* (*TCP-like*) and *SQUAMOSA promoter-binding-like* (*SPL-like*) genes (Hou and Yang, 2009; Chou et al., 2013; Lin et al., 2013, 2016; Jang, 2015; Liu et al., 2016). In association with transcription factors, microRNAs play crucial roles in flower development by regulating TF transcript levels. The miRNAs have been detected by deep-sequencing in orchids, such as *Phalaenopsis aphrodite*, *Erycina pusilla*, and *Dendrobium officinale* (An and Chan, 2012; Wang et al., 2013; Yang et al., 2015). However, little is known about how miRNAs function in orchid flower formation.

MiR396 is an evolutionarily conserved miRNA that recognizes a complementary sequence in the mRNA of land plant *GRF*s, encoding a plant-specific family of transcription factors involved in the control of cell proliferation. MiR396/*GRF* regulatory network affects plant growth and responses to environmental changes (Omidbakhshfard et al., 2015). In the model plant arabidopsis, *GRF*s targeted by miR396 regulate leaf and cotyledon growth, embryogenesis, and development of stem, flowers, and roots (Liebsch and Palatnik, 2020). Diverse functions of this module have also been described in non-model plants. For example, the miR396–GRF/GRF-interacting factor (GIF) module influences tomato (*Solanum lycopersicum*) fruit size (Cao D. et al., 2016), *Brassica* sp. root and leaf growth (Hong et al., 2018), maize (*Zea mays*) leaf size and plant height (Wu et al., 2014; Zhang et al., 2018), soybean (*Glycine max*) nematode resistance (Liu et al., 2017), and rice (*Oryza sativa*) plant height, meristem function, flowering time, inflorescence architecture, heading date, seed size and pathogen resistance (Gao et al., 2015; Sun et al., 2016; Shimano et al., 2018; Tang et al., 2018). MiR396–GRF module makes a crucial hub coordinating various growth and physiological responses with endogenous and environmental signals. This makes it a highly promising target for crop breeding and biotechnology. However, considering the extensive functional redundancy or sub-functionalization inherent to GRF family members resulting from sequence divergence and their distinct temporal and spatial expression patterns, the specific nature of underlying mechanisms remain unclear for many species.

The GRF family of TFs remains to be revealed in orchids. We therefore carried out the first comprehensive analysis and molecular dissection of *GRF* gene family in *C. ensifolium*, integrating comparative transcriptome analysis, gene structures, phylogenetic relationships, conserved protein motifs and expression patterns associated with plant development and flower ontogeny. We confirmed the existence of a conserved miR396–GRF module in orchids. We also examined the relationship between *Ce*-miR396 and *Ce*GRFs among different flower patterning varieties and their role in orchid flower development, revealing a large contribution to column specification. Transgenic *Nicotiana tabacum* overexpressing *Ce*-miR396 displayed curved pistil and reduced fertility. This study therefore illustrates that *CeGRFs* are regulated by *Ce*-miR396 during orchid flower development and provides building blocks for molecular breeding of orchids in the enhancement of their remarkable floral patterns.

## Materials and Methods

### Plant Materials and Growth Conditions

Wild-type plants and the natural mutants of *C. ensifolium* were artificially cultivated and collected from the cultivation base of Environmental Horticulture Research Institute, Guangdong Academy of Agricultural Sciences, China. All plants were grown and maintained in pots in a greenhouse at day/night temperatures of 26/23°C under 16-h light/8-h dark photoperiod.

### Library Construction and Illumina Sequencing

For transcriptome sequencing, we constructed independent cDNA libraries for sepal, petal, labellum, and gynostemium obtained from floral buds at developmental stage 3 of three individual plants, separately. The floral apex resembles an inverted triangle, and the ventral outer sepal and petals grow quickly in this stage. Two replications were included in each sample to create eight multiplexed cDNA libraries. The mRNAs were purified from total RNA using Oligotex mRNA Midi Kit (QIAGEN, Germany) and quantified using Nano-Drop 2000 spectrophotometer (Thermo Scientific, United States) to generate the cDNA library according to Illumina manufacturer’s instructions (Yang and Zhu, 2015). The purified mRNAs were fragmented to approximately 200 bp and subjected to first strand and second strand cDNA synthesis, followed by adaptor ligation and low-cycle enrichment using TruSeq^®^ RNA HT Sample Prep Kit (Illumina, United States). The purified library products were evaluated with Agilent 2200 TapeStation and Qubit^®^2.0 (Life Technologies, United States) and diluted to 10 pM for cluster generation *in situ* on the HiSeq3000 pair-end flow cell, followed by sequencing (2 × 100 bp). An average of more than 75 million reads were generated for each sample.

### Degradome Library Construction and Sequencing

The total RNA quantity and purity were analyzed using Bioanalyzer 2100 and RNA 6000 Nano Lab Chip Kit (Agilent, CA, United States) with RIN number > 7.0. Approximately 20 μg of total RNA was used to prepare Degradome library. We followed the method of German et al. (2008) with some modification. Briefly, about 150 ng of poly(A) + RNA was isolated annealed with Biotinylated Random Primers. The biotinylated RNA fragments were captured by streptavidin. The annealed products containing 5’-monophosphates were ligated to 5’ adaptor, followed by reverse transcription and PCR. Libraries were sequenced using the 5’ adapter only. Then, the single-end sequencing (36 bp) was performed on Illumina Hiseq2500 at the LC-BIO (Hangzhou, China).

### Analysis of Differentially Expressed Genes

Gene expression levels were calculated by FPKM values: FPKM = [total transcript fragments/mapped fragments (millions)] × transcript length (kb). Significant differences in gene expression between wild type and mutant were determined using edgeR. The false discovery rate (FDR) was applied to identify the threshold of *P*-value in multiple tests. An FDR < 0.05 and | log_2_ ratio| > 1 (two-fold change) were set as the threshold of significant difference in gene expression. Differentially expressed genes (DEGs) were annotated using gene ontology (GO) and Kyoto Encyclopedia of Genes and Genomes (KEGG) enrichment analyses (Yang and Zhu, 2015; Yang et al., 2017). All the DEGs were mapped to GO terms (or KEGG pathways) in the databases^1^ ^2^ and gene numbers were calculated for each term (or pathway) (Kanehisa and Goto, 2000; Conesa et al., 2005). Then a hypergeometric test was applied to find significantly enriched terms in DEGs compared to the genomic background. For this purpose, we used the corrected *P*-value ≤ 0.05 as a threshold for significantly enriched GO terms and *Q*-value ≤ 0.05 as a threshold for KEGG pathways.

### Protoplast Isolation and Transfection

The plasmid pAN580-*GFP* containing dual cauliflower mosaic virus 35S promoter and *GFP* gene was used as the empty control. The 124-bp sequence of *Ce*-miR396 precursor was cloned into vector pAN580-*GFP*, and replaced with the *GFP* gene, resulting in the plasmid pAN580-pre-*Ce*-miR396. The young leaves and flower buds at developmental stage 3 of *C. ensifolium* were collected for protoplast isolation. The protoplast isolation was conducted following the past protocols (Negrutiu et al., 1987). Petals were cut into 0.5–1.0 mm strips and transferred into the freshly prepared enzyme-solution [1.0% (weight/volume, *w*/*v*) Cellulase R10, 0.5% (*w*/*v*), Macerozyme R10, 500-mM D-mannitol, 20-mM KCl, and 20-mM MES (pH = 5.7), 10-mM CaCl_2_, 0.1% (*w*/*v*) BSA]. The released protoplasts were harvested after incubation at 28°C in the darkness with rotations of 30 rpm for 5–6 h. Protoplast transfection was carried out using PEG-mediated protocol with slight modifications. Briefly, an equal volume of freshly prepared PEG solution (40% (*w*/*v*) PEG 4000, 0.2 M mannitol and 0.1 M CaCl_2_) was gently mixed with plasmid DNA in MMG solution [15 mM MgCl_2_, 0.4 M mannitol and 4 mM MES (PH = 5.7)]. Transfected protoplasts were incubated at 23°C for 6–36 h in the darkness. Transfection efficiency was measured according to the expression of the GFP reporter of transient expression vector pAN580-GFP (Ren et al., 2020). The transfection efficiency was about 80% in both leaf- and flower-derived protoplasts. The GFP fluorescence was observed and 3–5 images were taken in random distribution under an LSM 710 confocal laser scanning microscope. Total RNA was extracted 0, 12, 24, and 36 h after transfection for reverse transcription followed by quantitative PCR (RT-qPCR), to analyze transcript abundance of *CeGRFs* and *Ce-*miR396 *in vivo*.

### Stem-Loop Reverse Transcription-PCR of miR396 and Reverse Transcription-Quantitative PCR of Target Growth-Regulating Factor Genes

The cDNA of mature miRNA was prepared using miRNA reverse transcription kit M-MLV (Takara, China), and the reverse-transcribed products were used as template for RT-qPCR with gene-specific primers. The miRNA specific stem-loop primers and gene-specific RT-qPCR primers were designed according to the protocol described previously (Chen et al., 2005). For target gene validation, the cleavage site-spanning fragments of GRF genes were detected. Total RNA extracted from different tissue types was reverse-transcribed by oligo (dT) primed cDNA synthesis protocol (Fermentas). The resulting cDNA was subjected to quantitative PCR using Bio-Rad CFX-96 RealTime PCR System (Bio-Rad, United States) in a final volume of 20 μl containing 2 μl of cDNA and 10 μl of SYBR premix Ex-taq™ (Takara, Japan). Ubiquitin was used as an internal control for normalization to compare gene expression level between the accessions. For each reported result, at least three independent biological samples were subjected to a minimum of three technical replicates. The primers designed with Primer 7.0 software are listed in Supplementary Table 1.

### Multiple Sequence Alignment and Phylogenetic Analysis

*Cymbidium ensifolium growth-regulating factor* coding sequences were identified in our transcriptome dataset using a Basic Local Alignment Tool (BLAST) analysis for proteins. A total of 92 complete protein sequences were aligned using MUSCLE, including 11 CeGRFs from *Cymbidium ensifolium*, 9 AtGRFs from *Arabidopsis thaliana*, 12 OsGRFs from *Oryza sativa*, 17 BrGRFs from *Brassica rapa*, 14 ZmGRFs from *Zea mays*, 10 DcGRFs from *Dendrobium catenatum*, 10 AsGRFs from *Apostasia shenzhenica*, and 9 PeGRFs from *Phalaenopsis equestris.* Based on alignment, a Maximum likelihood (ML) phylogenetic tree was constructed using MEGAX with 1,000 bootstrap replicates and Jones-Taylor-Thornton method (Kumar et al., 2016). The online software iTOL was applied to edit the phylogenetic tree.^3^ The conserved motifs of CeGRF proteins were analyzed online using Multiple Expectation Maximization for Motif Elicitation (MEME) with default parameters.^4^

### Plasmid Construction and Tobacco Transformation

We generated cDNAs for the precursor fold-back structure of *Ce*-miR396 by reverse transcription. After verification by sequencing, we inserted pre-*Ce*-miR396 into the pCambia1300 vector downstream of the constitutive Cauliflower Mosaic Virus 35S promoter. This construct was introduced into Agrobacterium tumefaciens strain GV3101 for transformation of tobacco by the leaf-disk method, as described in our previous work (Yang et al., 2009). Transgenic plants were screened on an MS medium containing 10 mg/L hygromycin, and 21 independent lines were obtained.

### Scanning Electron Microscopy

Dissected apices of mature flowers were fixed in a solution of 3% glutaraldehyde and 2% formaldehyde for 24 h. Samples were dehydrated in acetone, critical-point dried in liquid CO_2_, and mounted on stubs and sputter coated with 25 nm gold. Samples were examined using a JSM-6360LV (JEOL) scanning electron microscope.

## Results

### Ontogeny of *Cymbidium ensifolium* Flower Development

**FIGURE 1:**
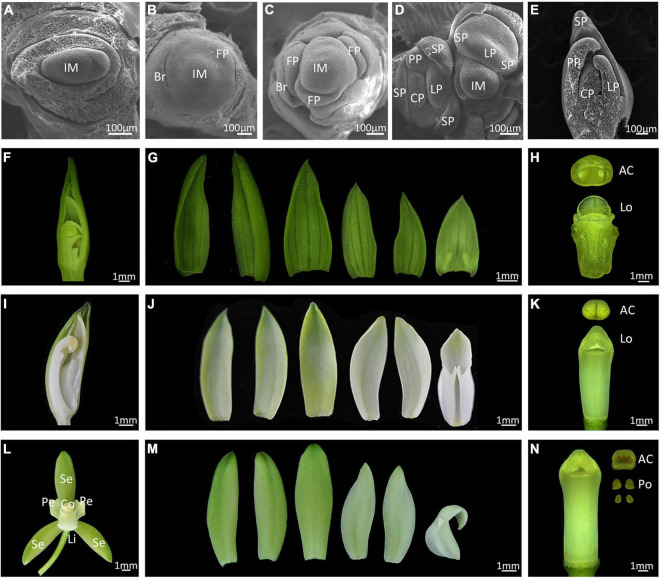
Floral development of *Cymbidium ensifolium.* **(A–E)** Scanning electron micrograph (SEM) of early floral developmental stages. Bar = 100 μm. Im, inflorescence meristem; Br, bract; FP, floret primordium; SP, sepal primordium; PP, petal primordium; LP, labellum primordium; CP, column primordium. **(F–N)** Developing flowers, Bar = 1 mm. The developing flower of stage 3 **(F–H)**, stage 4 **(I–K)**, and mature flowers **(L–N)**. Se, sepal; Pe, petal; Li, lip; Co, column; AC, Anther Cap; Lo, locule; Po, pollinium.

*Cymbidium ensifolium* commonly takes 3 years to reach reproductive maturity. Inflorescence meristems (IM) are produced in the peripheral regions of axillary buds and 6 to–11 flowers develop from each IM (Supplementary Figure 1). We divided floral development into six stages (from 0 to 5) according to visible changes in flower morphology (Figure 1). A crescent bract primordium initiates around the inflorescence meristem (Figure 1A), then the flower meristem emerges with a flattened and oval flower meristem (Figure 1B, stage 0), which continues to enlarge and form a floret primordium (FP) (Figure 1C, stage 1). A central transversal depression can be observed in FP, from which a labellum primordium (LP) initiates in the adaxial region, followed by development of two lateral sepal primordia (SP). The abaxial portion of primordia then enlarges, turning into two lateral petals and median sepal primordia. The column primordium finally emerges from the central region, establishing a typical orchid floral zygomorphy in this phase (Figures 1D,E, stage 2). This process is similar in the closely related species *C. sinense*, but develops much faster with a completion time of about 2 days (Su et al., 2018). During stage 3, the floral apex resembles an inverted triangle, with outer sepals overlapping the inner petals (Figures 1F,G). The ventral outer sepal and petals grow quickly and cover the column, which consists of an empty locule with no pollinia (Figure 1H). At stage 4, two ventral inner petals progressively shelter the labellum and column. The labellum develops a kinked to undulated margin, and the edge of labellum begins to curl toward column (Figures 1I,J). The column rapidly elongates at this stage but the pollinia are still not mature (Figure 1K). At stage 5, floral organ development completes with three petal-like sepals in the first whorl, two lateral petals and a specialized bottom petal (the lip or labellum) in the second whorl, and a fine-structured column in the central part (Figures 1L,M). The column includes carpel and stamens that are differentiated and evolved through the complete fusion of style, stigma, and staminal filament, and has four pollinia on a semi-circular viscidium (Figure 1N).

### Transcriptome Profiling

To identify key regulators controlling the specification and growth of individual floral organs, we isolated total RNA from each floral organ during stage 3. At this stage, all flower organ primordia differentiation is completed, enabling a comparative transcriptome analysis. From six pairwise comparisons between sepals, petals, labellum and gynostemium, we identified between 108 and 2,167 differentially-expressed unigenes in individual pairwise comparisons. A total of 3,017 unigenes exhibited significant changes in expression. The number of DEGs between tissues correlated with the degree of their morphological differences. The largest differences occurred between the gynostemium and sepals, with 1,324 up-regulated and 843 down-regulated transcripts. Moreover, 1,143 and 738 transcripts were differentially expressed in the gynostemium compared with petals (Supplementary Figure 2A). The smallest number of DEGs (108 transcripts) were found between sepals and petals. The number of DEGs in other pairwise comparisons ranged from 1,091 to 1,881 transcripts (Supplementary Table 2).

Gene Ontology (GO) enrichment analysis identified 41 GO terms significantly enriched among different floral organs. The main GO biological processes included oxidation-reduction and metabolism. The cellular components were mainly assigned to mitochondrion, plastids, and plasma membrane. The molecular functions predominantly involved metal ion binding and ATP binding (Supplementary Figure 2B and Supplementary Table 3). We identified 334 enriched KEGG pathways. The “metabolic and biosynthesis of secondary metabolites” pathway had the most DEGs, followed by “photosynthesis” and “plant hormone signal transduction” (Supplementary Table 4). Non-redundant (NR) annotation results indicated that most of the enriched transcripts were connected to plant metabolic processes, oxidation-reduction processes, and establishment of cell structures. We also observed a number of DEGs involved in secondary metabolism and oxidation-reduction pathways, such as genes encoding the enzymes, including acetyltransferase, transketolase, and NADP dependent oxidoreductase. Notably, many TFs among our DGEs; specifically, a large fraction (8 out of 11) of *GRF* family members were up-regulated in the inner whorls of flower (the column), suggesting a putative role in flower organ development and/or differentiation (Supplementary Figure 2C).

### Identification and Sequence Analysis of *Cymbidium ensifolium Growth-Regulating Factor* Genes

**FIGURE 2:**
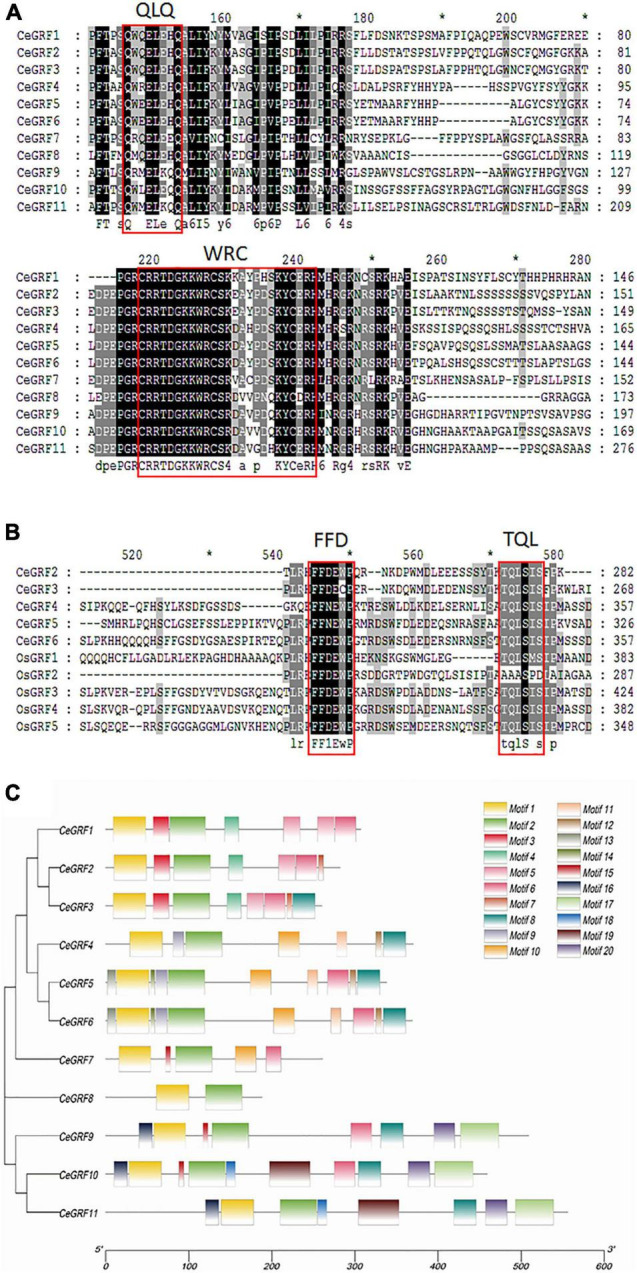
Sequence alignment and functional domain analysis of *Cymbidium ensifolium Growth-Regulating Factor* (CeGRF) proteins. The conserved QLQ and WRC domains in all CeGRFs **(A)** and the FFD and TQL motifs identified in CeGRF2, 3, 4, 5, and 6 **(B)** are indicated by the red box. Identical amino acids are indicated by black and the amino acids with >50% similarity are indicated by gray background. **(C)** Twenty conserved motifs labeled with different colors were identified in the CeGRF sequences using the MEME program. Among them, motif 1 and motif 2 are the QLQ and WRC conserved domains.

We identified 11 *CeGRFs* coding sequences in our transcriptome dataset. Considerable variation in length was present in their coding sequences, ranging from 468 to 1,788 bp. The deduced proteins varied between 155 and 595 amino acids (Supplementary Table 5). All of the putative CeGRF proteins shared the conserved QLQ and WRC domains in their N-terminal regions (Figure 2A). A zinc finger motif (CCCH) was also present within the WRC domains of all CeGRF proteins. Four CeGRFs (CeGRF2, CeGRF3, CeGRF4, CeGRF5, and CeGRF6) shared short stretches of amino acids termed FFD (phenylalanine, phenylalanine, and aspartate) and TQL (threonine, glutamine, and leucine) domains near their C-termini (Figure 2B).

The Multiple Em for Motif Elicitation (MEME) program predicted 20 distinct protein motifs in CeGRF proteins (Figure 2C). All CeGRFs contained motif 1 (QLQ motif) and motif 2 (WRC motif) at their N-terminus. Each CeGRF possessed 5–10 conserved motifs, except CeGRF8 which only possessed motifs 1 and 2 and showed an overall low sequence similarity with other GRFs. The closely related CeGRF1, CeGRF2, and CeGRF3 shared motifs 1–6; CeGRF2 presented domain 7 as well, while CeGRF3 had both motifs 7 and 8. The more distant CeGRF4, CeGRF5, and CeGRF6 contained the QLQ and WRC motifs, as well as motifs 8–14. The remaining CeGRF9, CeGRF10, and CeGRF11 contained additional motifs 15–20. Detailed information for each motif is listed in Supplementary Figure 3.

**FIGURE 3:**
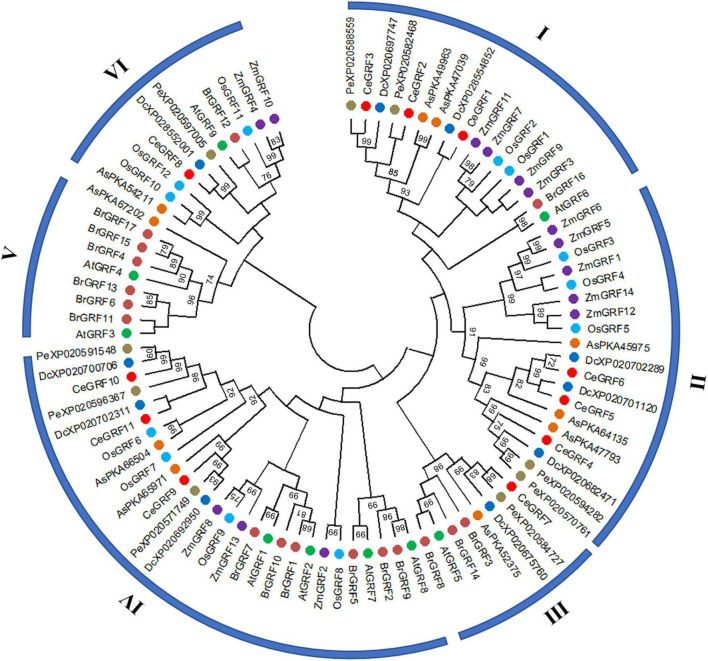
Phylogenetic relationships among GRFs. Neighbor-joining phylogenetic tree was constructed by MEGAX.0 software with the Poisson model and 1,000 bootstrap computations. The GRF proteins were grouped into six distinct types, and represented GRFs from *Arabidopsis thaliana* (At), *Oryza sativa* (*Os*), *Brassica rapa* (Br), *Zea mays* (Zm), *Dendrobium catenatum* (Dc), *Apostasia shenzhenica* (As) and *Phalaenopsis equestris* (Pe). Each species is represented by a different color.

We conducted a phylogenetic analysis of GRFs from different species to understand their evolutionary relationships. As shown in Figure 3 and Supplementary Figure 4, 92 GRFs clustered into six subgroups (I–VI) by the Maximum Likelihood phylogenetic tree method constructed by MEGA X. Subgroup II contained clusters of GRFs from monocot species only, while subgroup V was specific to dicot species only. CeGRFs were distributed across 5 out of 6 subgroups and were not represented in subgroup V, consistent with their classification as monocots. Subgroups III and V were relatively small, with only eight and nine GRFs each, respectively. By contrast, subgroup IV contained the largest number of GRFs (twenty-eight), followed by subgroups II (nineteen), I (seventeen), and VI (eleven). Notably, we found GRFs from orchid plants (*Cymbidium ensifolium, Dendrobium catenatum*, *Apostasia shenzhenica*, and *Phalaenopsis equestris*) within subgroups I, II, and III, which clustered away from monocot and dicot plants, indicating that GRFs are highly conserved between closely related orchid species and are clearly separated from other genera and more distant species.

### *Cymbidium ensifolium Growth-Regulating Factors* Are Regulated by *Cymbidium ensifolium*-miR396

**FIGURE 4:**
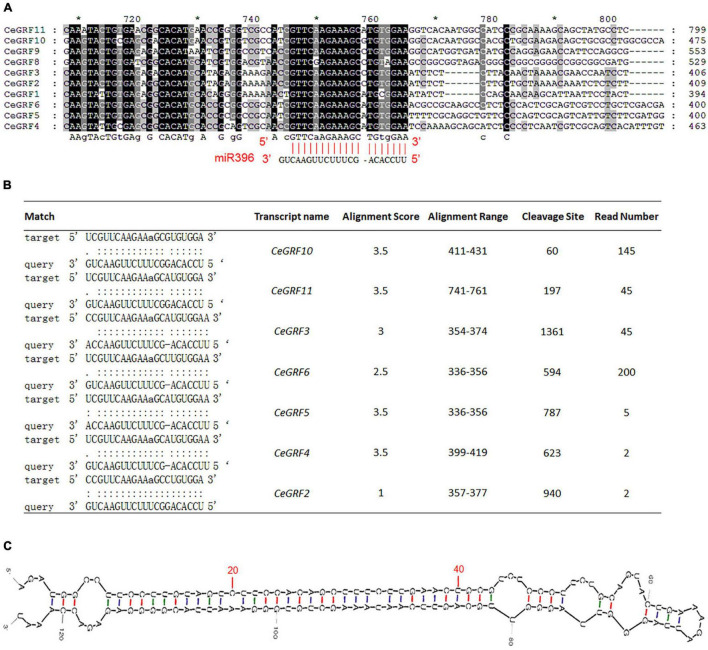
*Cymbidium ensifolium Growth-Regulating Factors* are Targeted by *Ce-*miR396. **(A)** Complementary matching between miR396 and *CeGRFs*. **(B)** Identification of the targeted GRF genes of miR396 by degradome sequencing, read number indicated normalized reads that matched the different positions of the targeted genes. Only the cleavage sites with *P* value < 0.05 were counted. **(C)** Secondary structure of pre-*Ce*-miR396 of *Cymbidium ensifolium*. The mature sequences that match the precursor detected by small RNA-Seq are displayed in red.

Arabidopsis *GRFs* are the predicted target genes for 21-nt microRNA miR396. *CeGRF* genes (except *CeGRF7*) share a highly similar sequence over the length of predicted miR396 complementary sequence, with only one mismatch and one bulge in the miR396 binding site (Figure 4A). Through degradome sequencing which is a powerful and efficient approach for the validation of miRNA-target genes, we observed that the transcripts of 7 out of 11 *CeGRFs* were cleaved by miR396 at the correct predicted position [the 11th nucleotide “A” of the target sequence “UCGUUCAAGAAaGCGUG(A)UGGA”] in *C. ensifolium*, with significant alignment scores between 1 and 3.5 and associated *p*-values ≤ 0.05. *CeGRF6* showed the largest normalized read counts out of all detected *CeGRFs*, followed by *CeGRF10*, indicating a strong *in vivo* signal (Figure 4B and Supplementary Table 6).

**FIGURE 5:**
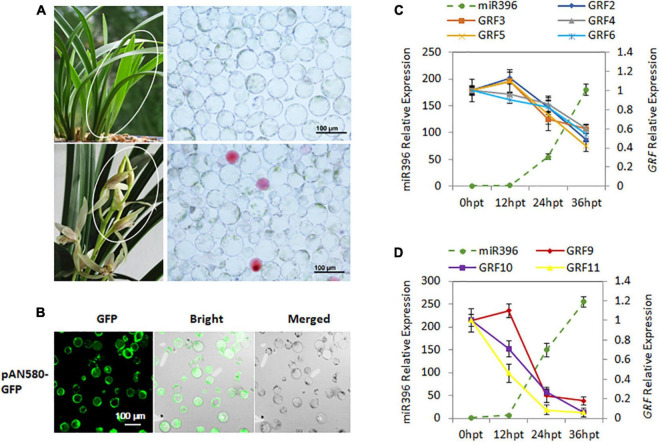
Validation of *Ce*-miR396-Regulated *CeGRFs* expression in a *C. ensifolium* Protoplast-Based Transient Expression System. **(A)** Protoplast isolation from the leaves (top) and flowers (bottom). **(B)** Efficient protoplast-based transient expression system (PTES) in *Cymbidium ensifolium*. The green fluorescence of the GFP reporter is clearly visible in most cells. The maximum transfection efficiency of pPAN-580-GFP (4.71 kb in size) was ∼80%. Bar = 100 μm. **(C,D)** Transcript levels of *Ce*-miR396 (dotted line) and *CeGRF* genes (solid lines) in leaves **(C)** and flowers **(D)**. Mature *Ce-*miR396 was successfully overexpressed in *Cymbidium* protoplasts 24–36 h after transfection, and the expression levels of *CeGRFs* were significantly reduced. The Y-axis indicates fold change in expression at different time points. Expression levels were normalized using the threshold cycle values obtained for the Ubiquitin gene. Error bars indicate the standard deviation of the mean (SD) (*n* = 3). Three replicates were analyzed, with similar results.

To further confirm the miR396-*GRF* regulatory module in *C. ensifolium*, we searched for *Ce*-miR396 precursor in our transcriptome dataset and identified a 124 bp sequence containing mature *Ce*-miR396 and complementary *Ce*-miR396* (Figure 4C). We overexpressed it in *C. ensifolium* using an improved and robust protoplast-based transient expression system (PTES) (Ren et al., 2020; Figures 5A,B). Mature *Ce-*miR396 was successfully overexpressed in *Cymbidium* protoplasts, increasing 150 to 350-fold within 36 h after transfection, which was accompanied by the down-regulated expression of *CeGRFs*, in agreement with the results from degradome sequencing. Notably, in leaf protoplasts, *CeGRF2*, *CeGRF3*, *CeGRF4*, *CeGRF5*, and *CeGRF6* transcript levels decreased significantly starting 24 h after transfection, reaching 40–60% in 36 h after transfection compared to time 0, while *CeGRF1*, *CeGRF9*, *CeGRF10*, and *CeGRF11* transcripts were not affected (Figure 5C). Contrarily, in flower protoplasts, the cleavage site-spanning fragments of *CeGRF9*, *CeGRF10*, or *CeGRF11* were almost undetectable, while no significant change in *CeGRF2*, *CeGRF3*, *CeGRF4*, *CeGRF5*, or *CeGRF6* expression was observed (Figure 5D and Supplementary Figure 5). Differential regulation of individual genes is likely due to different tissue-specific expression patterns of individual *CeGRF* genes.

### Expression Dynamics of *Cymbidium ensifolium-miR*396/*Cymbidium ensifolium Growth-Regulating Factor* Tightly Correlate With Reproductive Organ Development

**FIGURE 6:**
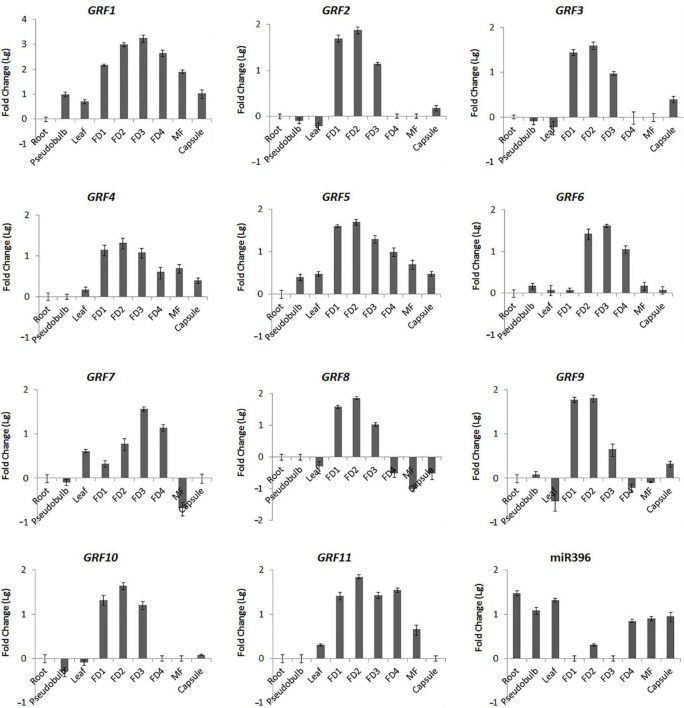
Organ-Specific Expression of *CeGRFs* and *Ce*-miR396. Expression of *CeGRF* genes and *Ce-*miR396 in different organs examined by Real-time RT-qPCR and stem-loop PCR. Tissues analyzed: roots, pseudobulb, leaves, five stages of Floral Development (FD 1, 2, 3, 4, and mature flower (MF). The Y-axis indicates fold change (Log value) in expression among different plant organs and the floral buds at different developmental stages. Expression levels were normalized using the threshold cycle values obtained for the Ubiquitin gene. Root and FD1 were used as a standard in presenting fold change (Lg) of *CeGRFs* and *Ce*-miR396, respectively. Error bars indicate the standard deviation of the mean (SD) (*n* = 3). Three replicates were analyzed, with similar results.

To validate the results obtained from DGEs and further explore the role of *Ce-*miR396/*CeGRF* in flower development, we analyzed their expression patterns in different floral developmental stages and organs by stem-loop (for *Ce*-miR396)- or RT (for *CeGRFs*)- qPCR. In general, all *CeGRF* genes were predominantly expressed in young buds (stages 1–4), reaching 15–2,500 fold higher levels than in other tissues, while *Ce-*miR396 was markedly low in these stages (Figure 6). The expression levels of *CeGRFs* were in general the lowest in roots, stems, and leaves, except for *CeGRF7* and *CeGRF*8, which showed the lowest expression levels in mature flowers. *CeGRF1*, *CeGRF4*, *CeGRF5*, and *CeGRF11* exhibited similar expression patterns and showed relatively high transcript levels in flowers, and the lowest levels in roots. *CeGRF2*, *CeGRF3*, *CeGRF8*, *CeGRF9*, *CeGRF10*, and *CeGRF11* were highly expressed during floral developmental stages 1–3. *CeGRF*6 and *CeGRF7* were more strongly expressed in floral developmental stages 2–4 compared to other tissues. By contrast, the expression of mature *Ce*-miR396 was low in developing floral buds and high in roots and leaves, generally showing the opposite expression pattern from *CeGRFs* (Figure 6).

**FIGURE 7:**
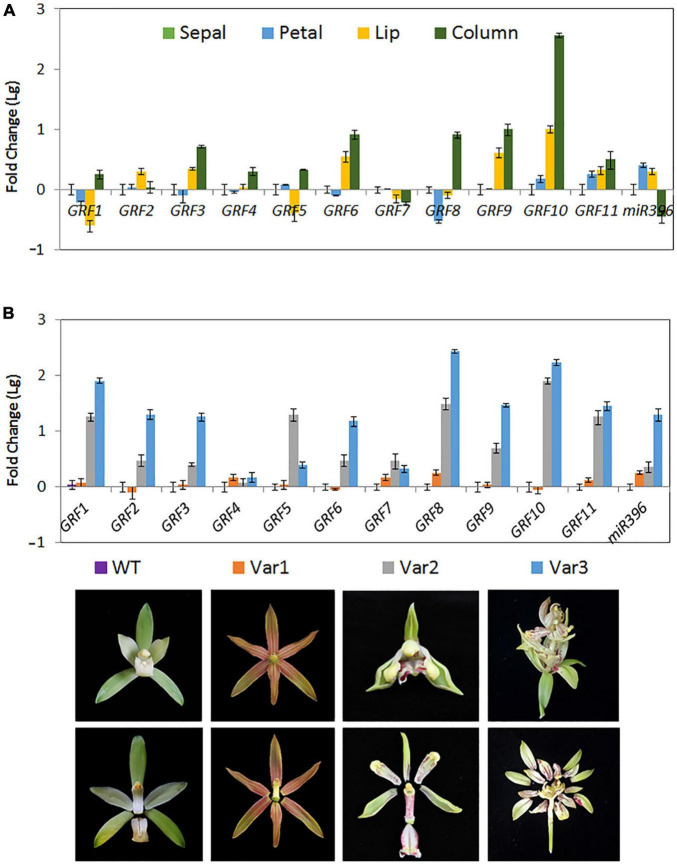
Expression of *Ce*-miR396/*CeGRF* Correlates with Flower Development. Expression of *CeGRF* genes and *Ce-*miR396 in different floral organs examined by RT-qPCR and stem-loop PCR, respectively. We analyzed individual floral organs (sepal, petal, lip, and column) **(A)** and the whole flowers of three floral varieties that develop non-lip (Var1) or stamenoid-tepal (Var2) or multi-tepal flowers (Var 3) **(B)**. The Y-axis indicates fold change (Log value) in expression among different floral organs. Expression levels were normalized using the threshold cycle values obtained for the Ubiquitin gene. The samples from sepal and the wild-type flower (WT) were used as a standard to present fold change (Lg). Error bars indicate the standard deviation of the mean (SD) (*n* = 3). Three replicates were analyzed, with similar results.

*Cymbidium ensifolium growth-regulating factors*, except *CeGRF7*, showed higher expression in inner whorls than in the other tissues (Figure 6). *CeGRF1*, *CeGRF4*, *CeGRF5*, and *CeGRF*8 were mainly expressed in the column. *CeGRF3*, *CeGRF6*, *CeGRF9*, and *CeGRF10* were highly expressed in the lip and column, reaching to 5–150 fold increase in expression, consistent with the high transcriptome expression of *CeGRFs* in the inner flower whorls. By contrast, *Ce-*miR396 showed relatively high expression in petal and sepal, and the lowest expression in column (Figure 7A). Thus, these results revealed a strong negative correlation between *Ce-*miR396 and *CeGRFs* transcript levels, with antagonistic distribution mainly in the column.

*Cymbidium ensifolium* evolves a number of natural variation types of flower organs according to floral organ transformation and/or reversion. In this study, three typical natural flower morphology mutants, which produce null-labellum flowers (Var1), stamenoid-like tepals (Var2), or multi-tepals (Var3), were employed to determine the relationship between *CeGRF* gene expression and flower development. When compared with standard flowers (ST), most *CeGRFs* (with the exception of *CeGRF4* and *CeGRF7*) were more strongly expressed in the varieties that developed stamenoid-tepal (Var 2) or multi-tepal (Var 3) flowers, with 2.5–270 fold increase in expression (Figure 7B). *Ce*-miR396 displayed the same trend (Figure 7B). However, reduced or comparable expression levels were detected in Var 1, which developed similar perianth structures with no lips or column, suggesting a strong correlation between *CeGRF* expression and reproductive organ development. By contrast, *CeGRF4* and *CeGRF7* had comparable expression levels in different floral organs, possibly reflecting functional differences among gene family members.

### Regulation of Flower Development by *Cymbidium ensifolium*-miR396 Overexpression in Tobacco

**FIGURE 8:**
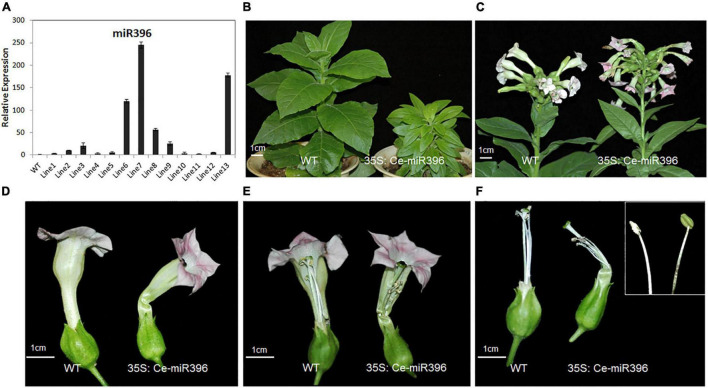
Phenotypes of Flowers Overexpressing Ce-*miR396* in Tobacco Plants. **(A)** Increased expression level of Ce-*miR396* in the transgenic lines. The Y-axis indicates fold change in expression between the wild-type and transgenic plants. Expression levels were normalized using the threshold cycle values obtained for the Ubiquitin gene. Error bars indicate the standard deviation of the mean (SD) (*n* = 3). Three replicates were analyzed, with similar results. **(B)** Reduced height and leaf size observed in transgenic plants. **(C–F)** Comparison of WT and transgenic flower inflorescence **(C)**, perianth **(D)**, pistils **(E)**, and filaments **(F)**. Bar = 1 cm.

To further dissect the function of *Ce-*miR396, we generated transgenic tobacco plants overexpressing *Ce*-miR396. We obtained 21 independent transgenic plants, a subset of which was analyzed for the presence of *Ce*-miR396. As shown in Figure 8A, All T_1_ plants had higher levels of *Ce*-miR396 ranging from 3.5 to 245 folds increase compared with wild type and empty construct. We further searched the tobacco genome sequence database and 41 putative *NtGRF* sequences were obtained. Among them, eight sequences with the highest identity with arabidopsis *GRFs* were selected for expression verification in wild-type and transgenic tobacco plants using quantitative RT-PCR (Supplementary Figure 6A). By amplifying the cleavage site-spanning fragment, we determined that, compared with WT, levels of eight *NtGRF-like* genes were significantly decreased in three independent transgenics lines 6, 7, and 8 (Supplementary Figure 6B), indicating a strong negative regulation of *NtGRF* transcript levels by *Ce*-miR396.

The transgenic plants were shorter and had small and narrow leaves compared to wild type (Figure 8B). These phenotypes resembled those seen in the *grf1 grf2 grf3* triple mutant in arabidopsis and transgenic tobacco plants overexpressing arabidopsis miR396 (Kim and Kende, 2004), further supporting a conserved role of *Ce*-miR396 reflected by this narrow-leaf phenotype. The perianth of transgenic plants was similar in structure with that of wild type but was severely curved (Figures 8C,D). The stamens were shorter and some of the anthers developed abnormally with no pollen. Even when the anthers properly released their pollen, stamens were much shorter than pistils, leading to partial sterility, which is similar to the phenotypes associated with arabidopsis lines overexpressing miR396 (Figures 8E,F).

## Discussion

### Expression Patterns of Growth-Regulating Factor Genes in *Cymbidium ensifolium*

Orchid floral development is a hot research topic. We previously published the analysis of 111,892 *C. ensifolium* transcript clusters derived from *de novo* assembly of flower transcriptome (Yang and Zhu, 2015). In the present study, we dissected the *C. ensifolium* flower ontogeny for the first time, and identified 3,017 DEGs by comparative transcriptome analysis among different floral organs. Most DEGs were involved in plant metabolic processes, oxidation-reduction, plant hormone signaling and establishment of cell structures. These are related to the developmental processes underlying flower development, i.e., cell division, membrane-building, and regulation of anabolism. We also identified a number of TFs from several families, including the well-known floral-related MADS-box, NAC, NF-YC, TALE, as well as bZIP, bHLH, FAR1, Zn-finger, and MYB, highlighting the importance of TFs in organ-specific development (Supplementary Table 7). Notably, we observed significant expression of most *CeGRF* genes in the column, signifying the possible role of *CeGRFs* in *C. ensifolium* flower development.

Investigating tissue specificity in expression patterns can provide clues to tissue development. Indeed, *GRF*s are expressed mainly in specific organs and tissues. For example, in *Medicago truncatula*, eight *GRF* genes exhibited lower expression in leaves compared with their expression in other tissues of roots, shoots, and flowers (Bazin et al., 2013). In Chinese cabbage, seven (out of 17) *BrGRF* genes showed higher expression in young leaves compared to other tissues (Wang et al., 2014). In cucumber (*Cucumis sativus*), *CsGRFs* were highly transcribed in ovaries, although *CsGRF4* and *CsGRF6* exhibited the highest expression in leaves, and *CsGRF7* was only detected in roots (Shi et al., 2019). Collectively, these studies highlight the tissue-specific expression diversity of *GRFs* in various plant species (Cao Y. et al., 2016).

Compared to *GRFs* in other plants, our results showed that 10 out of the 11 *CeGRF* genes were predominantly expressed in fast-growing floral buds. In addition, we detected much higher expression of *CeGRF* genes in the column of both wild type and varieties with an over-developed column (Var 2 and Var3, Figure 7B), and a converse lower expression in Var1 with no column. These results reveal the important role of *GRFs* in flower development, specifically in column formation and growth. Moreover, it is worth noting that genes belonging to the same phylogenetic groups displayed a similar expression pattern, for example *CeGRF2*/*CeGRF3* in subgroup I, *CeGRF4*/*CeGRF5* in subgroup II, and *CeGRF9*/*CeGRF10* in subgroup IV (Figure 3). This suggests that members of same group, sharing the same functional domains, play similar functions in flower development.

### Different Spatio-Temporal Activity of *Cymbidium ensifolium-miR*396/*Cymbidium ensifolium Growth-Regulating Factors* in *Cymbidium ensifolium*

MiR396 is an evolutionarily conserved miRNA that recognizes a complementary sequence in *GRF* genes. The components of this miR396–GRF module are conserved in seed plants and work antagonistically to affect the development of various organs in many species. A few examples of development and responses mediated by the miR396–GRF module include floral organ growth, development and fertility (rice and maize), seed and grain size (rice, arabidopsis, *Brassica napus*, and tomato), shoot and inflorescence growth (arabidopsis, tomato, rice, and maize), flowering time (arabidopsis and rice), pathogen resistance (Arabidopsis and rice), photosynthetic activity (arabidopsis and rice), senescence delay arabidopsis drought tolerance (arabidopsis), root growth (rice), leaf angle (rice and maize) and embryogenic transition (arabidopsis) (Kim et al., 2003, 2012; Horiguchi et al., 2005; Luo et al., 2005; Kuijt et al., 2014; Li et al., 2016; Kim, 2019).

This clearly illustrates that miR396 and *GRFs* may have different regulatory roles in different species during plant development, as a consequence of different activity, over time and development, of the downstream target genes. In our study, *CeGRFs* were regulated differently in different organs. When *Ce*-miR396 was overexpressed in a *C. ensifolium*, a subset of *CeGRFs* (*CeGRF2*, *CeGRF3*, *CeGRF4*, *CeGRF5*, and *CeGRF6*) behaved similarly in leaf protoplasts, exhibiting a similar expression pattern and *Ce*-miR396-mediated cleavage, lowering their transcript levels up to 40–60%. The same *CeGRFs* were, however, not affected by *Ce*-miR396 overexpression in flower protoplasts. By contrast, *CeGRF9*, *CeGRF10* and *CeGRF11* exhibited the opposite behavior. The transcript levels of *CeGRF1* and *CeGRF7* showed no significant differences in either case. These results indicate the functional diversity of individual members. In addition, *CeGRF9*, *CeGRF10*, and *CeGRF11* may play important roles in flower development, under the regulation of *Ce*-miR396.

### Floral Development Regulation of *Cymbidium ensifolium* by *Cymbidium ensifolium-miR*396/*Cymbidium ensifolium Growth-Regulating Factor*

The miR396/*GRF* module in both eudicots and monocots plays essential roles in the growth and development of floral organs, although the specific mechanism remains unclear for many species. Detailed developmental studies of various higher order loss-of-function mutants like *grf1 grf 2 grf 3* and associated co-activators *gif1 gif 2 gif 3*, as well as *gif* mutants overexpressing miR396 in arabidopsis have uncovered the individual contribution of specific family member in reproductive development (Liang et al., 2014). Arabidopsis GIFs are essential factors for carpel and stamen development, regulating the formation and maintenance of meristematic structures of male and female reproductive organs. In addition, the functionality of the miR396/*GRF* module is conserved in eudicots floral organ development, causing aberrant floral organs reminiscent of *grf* and *gif* mutant phenotypes (Kim and Tsukaya, 2015; Lee et al., 2018).

However, this conservation does not extend to monocots, because the aberrant floral development caused by miR396/GRF in monocots is morphologically distinct from that of eudicots (Luo et al., 2005). Overexpression of rice miR396, or double mutants in the rice *GRF6* and *GRF10* genes produced open husks, long sterile lemmas, and/or anomalous stigma and anther numbers (Duan et al., 2015). The maize *gif1* mutant produced sterile male and female flowers, with multiple silks (pistils) per floret and nucellus protruding out of the carpel (Wu et al., 2014; Zhang et al., 2018). This diversity of phenotypes was explained by the interaction of GIF with tissue-specific GRFs, leading to the regulation of distinct downstream targets. A complementary mechanism may also call upon the regulation of *GRFs* by different miR396 loci. For example, in arabidopsis, overexpression of miR396a causes a more severe floral organ defect phenotype than that of miR396b. Notably, the TF HECATE1 (Gremski et al., 2007), which controls carpel development in arabidopsis, is a direct target gene of miR396/*GRF* module, whereby *GRF* levels influence floral organ growth and development. In rice, *Os*-miRNA396d and its targets *Os*GRF6 and *Os*GRF10, together with *Os*GIF1, are involved in the regulation of floral organ development through Jumonji C domain-containing histone demethylase *Os*JMJ706 and the crinkly4 receptor-like kinase OsCR4 (Liu et al., 2014).

In this study, we conclude that *Ce-*miR396 and *CeGRF9*, *CeGRF10*, and *CeGRF11* are involved in the regulation of flower development based on: the positive correlation between gene expression and phenotypes, target gene cleavage by *Ce*-miR396 in flower resulting in decreased *CeGRF* transcript levels, and the phenotypes caused by *Ce*-miR396 overexpression in tobacco. Considering the preferential expression of *CeGRF9*, *CeGRF10*, and *CeGRF11* in the column of wild type and varieties with over-development of column, and their lower expression in a variety lacking the column structure, we hypothesize that *CeGRF9*, *CeGRF10*, and *CeGRF11* function in column development. Notably, *CeGRF9*, *CeGRF10*, and *CeGRF11* belongs to subgroup V and clustered closely with genes that were reported to function in floral development in other plants, such as arabidopsis *GRF1*, *GRF2*, and *GRF3*, as well as rice *GRF6*. This indicates a conserved role across eudicot and monocot species despite the different consequences of their respective overexpression, such as open husks for *Os*GRF6, or fused or curved flowers for arabidopsis GRF1, GRF 2, and GRF3. However, the orchid floral organs, and especially the column, are unique floral structures that may indicate a shift in protein functions and interactions in floral homeotic genes (Mondragón-Palomino and Theißen, 2008; Hsu et al., 2015). In this sense, screening of target genes downstream of GRFs and their co-activator GIF will further extend our knowledge of the regulation of *Ce*-miR396/*Ce*GRF in orchids flower development.

## Data Availability Statement

The datasets presented in this study can be found in online repositories. The names of the repository/repositories and accession number(s) can be found in the article/Supplementary Material.

## Author Contributions

FY and GZ designed the experiments and wrote the manuscript with input from all authors. GL and YW analyzed the data. JJ and CL executed the experiments and assembled the figures. JW conducted the qRT-PCR. SA and YX edited the manuscript. All authors read and approved the final manuscript.

## Conflict of Interest

The authors declare that the research was conducted in the absence of any commercial or financial relationships that could be construed as a potential conflict of interest.

## Publisher’s Note

All claims expressed in this article are solely those of the authors and do not necessarily represent those of their affiliated organizations, or those of the publisher, the editors and the reviewers. Any product that may be evaluated in this article, or claim that may be made by its manufacturer, is not guaranteed or endorsed by the publisher.

## Footnotes

1
http://www.geneontology.org/

2
http://www.genome.ad.jp/

3
http://itol.embl.de/

4
http://meme-suite.org/tools/meme

## Supplementary Material

Supplementary Figure 1 Floral development process of *Cymbidium ensifolium.*

Supplementary Figure 2 Comparative transcriptome analysis of individual floral Organs. **(A)** Transcripts differentially expressed between different floral organs. Up- and down-regulated transcripts were quantified. The results of six comparisons between each two samples are shown. Se, sepal; Pe, petal; Li, Lip; Co, column. **(B)** GO term classification of differentially-expressed unigenes. **(C)** Expression heat map of *CeGRF* genes in sepal, petal, Lip, and column. Expression values from RNA-seq data were log_2_-transformed and are displayed as filled blocks in blue to red indicating gene expression intensity from low to high.

Supplementary Figure 3 Detailed information for protein motifs predicted in CeGRFs.

Supplementary Figure 4 Phylogenetic analysis of CeGRFs and AtGRFs.

Supplementary Figure 5 *Cymbidium ensifolium*-miR396-regulated *CeGRFs* expression in a *Cymbidium ensifolium* protoplast-based transient expression system.

Supplementary Figure 6 *Cymbidium ensifolium*-miR396-regulated *NtGRFs* expression in transgenic tobacco plants.

## Abbreviations

GRF: growth regulating factor
GIF: GRF-interacting factor
DEGs: differentially expressed genes
GO: gene ontology
KEGG: Kyoto encyclopedia of genes and genomes
TF: transcription factor
PTES: protoplast-based transient expression system
NF-Y: nuclear factor Y
TALE: three-amino-acid-loop-extension
Bzip: basic leucine zipper domain
bHLH: basic helix-loop-helix.

## References

[B1] Aceto S. Gaudio L. (2011). The MADS and the Beauty: Genes Involved in the Development of Orchid Flowers. *Curr. Genomics* 12 342–356. 10.2174/138920211796429754 22294877 PMC3145264

[B2] An F. M. Chan M. T. (2012). Transcriptome-wide characterization of miRNA-directed and non-miRNA-directed endonucleolytic cleavage using Degradome analysis under low ambient temperature in *Phalaenopsis aphrodite* subsp. formosana. *Plant Cell Physiol.* 53 1737–1750. 10.1093/pcp/pcs118 22904110

[B3] Bazin J. Khan G. A. Combier J. P. Bustos-Sanmamed P. Debernardi J. M. Rodriguez R. (2013). miR396 affects mycorrhization and root meristem activity in the legume *Medicago truncatula*. *Plant J.* 74 920–934. 10.1111/tpj.12178 23566016

[B4] Cao D. Wang J. Ju Z. Liu Q. Li S. Tian H. (2016). Regulations on growth and development in tomato cotyledon, flower and fruit via destruction of miR396 with short tandem target mimic. *Plant Sci.* 247 1–12. 10.1016/j.plantsci.2016.02.012 27095395

[B5] Cao Y. Han Y. Jin Q. Lin Y. Cai Y. (2016). Comparative Genomic Analysis of the GRF Genes in Chinese Pear (*Pyrus bretschneideri Rehd*), Poplar (*Populous*), Grape (*Vitis vinifera*), Arabidopsis and Rice (*Oryza sativa*). *Front. Plant Sci.* 7:1750. 10.3389/fpls.2016.01750 27933074 PMC5121280

[B6] Chen C. Ridzon D. A. Broomer A. J. Zhou Z. Lee D. H. Nguyen J. T. (2005). Real-time quantification of microRNAs by stem-loop RT-PCR. *Nucleic Acids* R*es.* 33 e179–e179. 10.1093/nar/gni178 16314309 PMC1292995

[B7] Chou M. L. Shih M. C. Chan M. T. Liao S. Y. Hsu C. T. Haung Y. T. (2013). Global transcriptome analysis and identification of a CONSTANS-like gene family in the orchid *Erycina pusilla*. *Planta* 237 1425–1441. 10.1089/dna.2014.2469 23417646

[B8] Conesa A. Gotz S. Garcia-Gomez J. M. Terol J. Talon M. Robles M. (2005). Blast2GO: a universal tool for annotation, visualization and analysis in functional genomics research. *Bioinformatics* 21 3674–3676. 10.1093/bioinformatics/bti610 16081474

[B9] Duan P. Ni S. Wang J. Zhang B. Xu R. Wang Y. (2015). Regulation of OsGRF4 by OsmiR396 controls grain size and yield in rice. *Nat. Plants* 2:15203. 10.1038/nplants.2015.203 27250749

[B10] Gao F. Wang K. Liu Y. Chen Y. Chen P. Shi Z. (2015). Blocking miR396 increases rice yield by shaping inflorescence architecture. *Nat. Plants* 2:15196. 10.1038/nplants.2015.196 27250748

[B11] German M. A. Pillay M. Jeong D. H. Hetawal A. Luo S. Janardhanan P. (2008). Global identification of microRNA-target RNA pairs by parallel analysis of RNA ends. *Nat. Biotechnol.* 26 941–946. 10.1038/nbt1417 18542052

[B12] Givnish T. J. Spalink D. Ames M. Lyon S. P. Hunter S. J. Zuluaga A. (2015). Orchid phylogenomics and multiple drivers of their extraordinary diversification. *Proc. Biol. Sci.* 282:1553. 10.1098/rspb.2015.1553 26311671 PMC4571710

[B13] Gremski K. Ditta G. Yanofsky M. F. (2007). The HECATE genes regulate female reproductive tract development in *Arabidopsis thaliana*. *Development* 134 3593–3601. 10.1242/dev.011510 17855426

[B14] Hong J. K. Suh E. J. Lee S.-B. Yoon H.-J. Lee Y.-H. (2018). Effects of Over expression of *Brassica rapa* GROWTH-REGULATING FACTOR Genes on *B. napus* Organ Size. *Korean J. Breed Sci.* 50 378–386.

[B15] Horiguchi G. Kim G. T. Tsukaya H. (2005). The transcription factor AtGRF5 and the transcription coactivator AN3 regulate cell proliferation in leaf primordia of *Arabidopsis thaliana*. *Plant J.* 43 68–78. 10.1111/j.1365-313X.2005.02429.x 15960617

[B16] Hou C. J. Yang C. H. (2009). Functional analysis of FT and TFL1 orthologs from orchid (*Oncidium Gower Ramsey*) that regulate the vegetative to reproductive transition. *Plant Cell Physiol.* 50 1544–1557. 10.1093/pcp/pcp099 19570813

[B17] Hsu H.-F. Hsu W.-H. Lee Y.-I. Mao W.-T. Yang J.-Y. Li J.-Y. (2015). Model for perianth formation in orchids. *Nat. Plants* 1:15046. 10.1038/nplants.2015.46

[B18] Jang S. (2015). Functional Characterization of PhapLEAFY, a FLORICAULA/LEAFY Ortholog in *Phalaenopsis aphrodite*. *Plant Cell Physiol.* 56 2234–2247. 10.1093/pcp/pcv130 26493518

[B19] Kanehisa M. Goto S. (2000). KEGG: kyoto encyclopedia of genes and genomes. *Nucleic Acids Res.* 28 27–30. 10592173 10.1093/nar/28.1.27 PMC102409

[B20] Kim J. H. (2019). Biological roles and an evolutionary sketch of the GRF-GIF transcriptional complex in plants. *BMB Rep.* 52 227–238. 10.5483/BMBRep.2019.52.4.051 30885290 PMC6507847

[B21] Kim J. H. Choi D. Kende H. (2003). The AtGRF family of putative transcription factors is involved in leaf and cotyledon growth in Arabidopsis. *Plant J.* 36 94–104. 10.1046/j.1365-313X.2003.01862.x 12974814

[B22] Kim J. H. Kende H. (2004). A transcriptional coactivator, AtGIF1, is involved in regulating leaf growth and morphology in Arabidopsis. *Proc. Natl. Acad. Sci. U S A* 101 13374–13379. 10.1073/pnas.0405450101 15326298 PMC516574

[B23] Kim J. H. Tsukaya H. (2015). Regulation of plant growth and development by the growth-regulating factor and grf-interacting factor duo. *J. Exp. Bot.* 66 6093–6107. 10.1093/jxb/erv349 26160584

[B24] Kim J. S. Mizoi J. Kidokoro S. Maruyama K. Nakajima J. Nakashima K. (2012). Arabidopsis growth-regulating factor7 functions as a transcriptional repressor of abscisic acid- and osmotic stress-responsive genes, including DREB2A. *Plant Cell* 24 3393–3405. 10.1105/tpc.112.100933 22942381 PMC3462639

[B25] Kuijt S. J. H. Greco R. Agalou A. Shao J. Hoen C. C. J. Overnas E. (2014). Interaction between the GROWTH-REGULATING FACTOR and KNOTTED1-LIKE HOMEOBOX Families of Transcription Factors. *Plant Physiol.* 164 1952–1966. 10.1104/pp.113.222836 24532604 PMC3982755

[B26] Kumar S. Stecher G. Tamura K. (2016). MEGA7: Molecular Evolutionary Genetics Analysis Version 7.0 for Bigger Datasets. *Mol Biol Evol* 33 1870–1874. 10.1093/molbev/msw054 27004904 PMC8210823

[B27] Lee S. J. Lee B. H. Jung J. H. Park S. K. Song J. T. Kim J. H. (2018). Growth-regulating factor and grf-interacting factor specify meristematic cells of gynoecia and anthers. *Plant Physiol.* 176 717–729. 10.1104/pp.17.00960 29114079 PMC5761776

[B28] Li J. Zhu G. F. Wang Z. H. (2017). Chemical Variation in Essential Oil of *Cymbidium sinense* Flowers from Six Cultivars. *J. Essent Bear Pl.* 20 385–394. 10.1080/0972060X.2017.1311236

[B29] Li S. Gao F. Xie K. Zeng X. Cao Y. Zeng J. (2016). The OsmiR396c-OsGRF4-OsGIF1 regulatory module determines grain size and yield in rice. *Plant Biotechnol. J.* 14 2134–2146. 10.1111/pbi.12569 27107174 PMC5095787

[B30] Liang G. He H. Li Y. Wang F. Yu D. (2014). Molecular mechanism of microRNA396 mediating pistil development in Arabidopsis. *Plant Physiol.* 164 249–258. 10.1104/pp.113.225144 24285851 PMC3875806

[B31] Liebsch D. Palatnik J. F. (2020). MicroRNA miR396, GRF transcription factors and GIF co-regulators: a conserved plant growth regulatory module with potential for breeding and biotechnology. *Curr. Opin. Plant Biol.* 53 31–42. 10.1016/j.pbi.2019.09.008 31726426

[B32] Lin C. S. Chen J. J. Huang Y. T. Hsu C. T. Lu H. C. Chou M. L. (2013). Catalog of *Erycina pusilla* miRNA and categorization of reproductive phase-related miRNAs and their target gene families. *Plant Mol. Biol.* 82 193–204. 10.1007/s11103-013-0055-y 23575662

[B33] Lin Y. F. Chen Y. Y. Hsiao Y. Y. Shen C. Y. Hsu J. L. Yeh C. M. (2016). Genome-wide identification and characterization of TCP genes involved in ovule development of Phalaenopsis equestris. *J. Exp. Bot.* 67 5051–5066. 10.1093/jxb/erw273 27543606 PMC5014156

[B34] Liu H. Guo S. Xu Y. Li C. Zhang Z. Zhang D. (2014). OsmiR396d-Regulated OsGRFs Function in Floral Organogenesis in Rice through Binding to Their Targets OsJMJ706 and OsCR4. *Plant Physiol.* 165 160–174. 10.1104/pp.114.235564 24596329 PMC4012577

[B35] Liu W. Zhou Y. Li X. Wang X. Dong Y. Wang N. (2017). Tissue-Specific Regulation of Gma-miR396 Family on Coordinating Development and Low Water Availability Responses. *Front. Plant Sci.* 8:1112. 10.3389/fpls.2017.01112 28694817 PMC5483475

[B36] Liu X. R. Pan T. Liang W. Q. Gao L. Wang X. J. Li H. Q. (2016). Overexpression of an Orchid (*Dendrobium nobile*) SOC1/TM3-Like Ortholog, DnAGL19, in Arabidopsis Regulates HOS1-FT Expression. *Front. Plant Sci.* 7:99. 10.3389/fpls.2016.00099 26904066 PMC4746357

[B37] Luo A.-D. Liu L. Tang Z.-S. Bai X.-Q. Cao S.-Y. Chu C.-C. (2005). Down-Regulation of OsGRF1 Gene in Rice rhd1 Mutant Results in Reduced Heading Date. *J. Integr. Plant Biol.* 47 745–752. 10.1111/j.1744-7909.2005.00071.x

[B38] Mondragón-Palomino M. Theißen G. (2008). MADS about the evolution of orchid flowers. *Trends Plant Sci.* 13 51–59. 10.1016/j.tplants.2007.11.007 18262819

[B39] Negrutiu I. Shillito R. Potrykus I. Biasini G. Sala F. (1987). Hybrid genes in the analysis of transformation conditions : I. Setting up a simple method for direct gene transfer in plant protoplasts. *Plant Mol. Biol.* 8 363–373. 10.1007/BF00015814 24301258

[B40] Omidbakhshfard M. A. Proost S. Fujikura U. Mueller-Roeber B. (2015). Growth-Regulating Factors (GRFs): A Small Transcription Factor Family with Important Functions in Plant Biology. *Mol. Plant* 8 998–1010. 10.1016/j.molp.2015.01.013 25620770

[B41] Ramirez S. R. Gravendeel B. Singer R. B. Marshall C. R. Pierce N. E. (2007). Dating the origin of the Orchidaceae from a fossil orchid with its pollinator. *Nature* 448 1042–1045. 10.1038/nature06039 17728756

[B42] Ren R. Gao J. Lu C. Wei Y. Jin J. Wong S. M. (2020). Highly Efficient Protoplast Isolation and Transient Expression System for Functional Characterization of Flowering Related Genes in *Cymbidium* Orchids. *Int. J. Mol. Sci.* 21:21072264. 10.3390/ijms21072264 32218171 PMC7177621

[B43] Shi Y. Liu H. Gao Y. Wang Y. Wu M. Xiang Y. (2019). Genome-wide identification of growth-regulating factors in moso bamboo (*Phyllostachys edulis*): in silico and experimental analyses. *Plant Omics* 7:e7510. 10.7717/peerj.7510 31579567 PMC6769349

[B44] Shimano S. Hibara K. I. Furuya T. Arimura S. I. Tsukaya H. Itoh J. I. (2018). Conserved functional control, but distinct regulation, of cell proliferation in rice and Arabidopsis leaves revealed by comparative analysis of GRF-INTERACTING FACTOR 1 orthologs. *Development* 145:159624. 10.1242/dev.159624 29567670

[B45] Su S. Shao X. Zhu C. Xu J. Lu H. Tang Y. (2018). Transcriptome-Wide Analysis Reveals the Origin of Peloria in Chinese Cymbidium (*Cymbidium sinense*). *Plant Cell Physiol.* 59 2064–2074. 10.1093/pcp/pcy130 29986119

[B46] Sun P. Zhang W. Wang Y. He Q. Shu F. Liu H. (2016). OsGRF4 controls grain shape, panicle length and seed shattering in rice. *J. Integr. Plant Biol.* 58 836–847. 10.1111/jipb.12473 26936408 PMC5089622

[B47] Tang Y. Liu H. Guo S. Wang B. Li Z. Chong K. (2018). OsmiR396d Affects Gibberellin and Brassinosteroid Signaling to Regulate Plant Architecture in Rice. *Plant Physiol.* 176 946–959. 10.1104/pp.17.00964 29180380 PMC5761777

[B48] Wang F. Qiu N. Ding Q. Li J. Zhang Y. Li H. (2014). Genome-wide identification and analysis of the growth-regulating factor family in Chinese cabbage (Brassica rapa L. ssp. pekinensis). *BMC Genomics* 15:807. 10.1186/1471-2164-15-807 25242257 PMC4180144

[B49] Wang H. H. Wonkka C. L. Treglia M. L. Grant W. E. Smeins F. E. Rogers W. E. (2015). Species distribution modelling for conservation of an endangered endemic orchid. *AoB Plants* 7:39. 10.1093/aobpla/plv039 25900746 PMC4463238

[B50] Wang J. Zhang C. Yan Y. Wu W. Ma Z. (2013). Identification of conserved MicroRNAs and their targets in Phalaenopsis orchid. *Russ. J. Plant Physiol.* 60 845–854. 10.1134/S1021443713060150

[B51] Wu L. Zhang D. Xue M. Qian J. He Y. Wang S. (2014). Overexpression of the maize GRF10, an endogenous truncated growth-regulating factor protein, leads to reduction in leaf size and plant height. *J. Integr. Plant Biol.* 56 1053–1063. 10.1111/jipb.12220 24854713

[B52] Xiang L. Chen Y. Chen L. Fu X. Zhao K. Zhang J. (2018). B and E MADS-box genes determine the perianth formation in *Cymbidium goeringii* Rchb.f. *Physiol. Plant* 162 353–369. 10.1111/ppl.12647 28967227

[B53] Yang F. Gang L. Liu D. Yu Di. (2009). Arabidopsis MiR396 Mediates the Development of Leaves and Flowers in Transgenic Tobacco. *J. Plant Biol.* 52 475–481. 10.1007/s12374-009-9061-7

[B54] Yang F. Zhu G. (2015). Digital Gene Expression Analysis Based on De Novo Transcriptome Assembly Reveals New Genes Associated with Floral Organ Differentiation of the Orchid Plant *Cymbidium ensifolium*. *PLoS One* 10:e0142434. 10.1371/journal.pone.0142434 26580566 PMC4651537

[B55] Yang F. Zhu G. Wang Z. Liu H. Xu Q. Huang D. (2017). Integrated mRNA and microRNA transcriptome variations in the multi-tepal mutant provide insights into the floral patterning of the orchid *Cymbidium goeringii*. *BMC Genomics* 18:367. 10.1186/s12864-017-3756-9 28490318 PMC5426072

[B56] Yang Z. Yang D. Ding X. Gao Y. Li D. Xu T. (2015). MicroRNA expression profiles in conventional and micropropagated *Dendrobium officinale*. *Genes Genomics* 37 315–325. 10.1007/s13258-014-0257-y

[B57] Zhang D. Sun W. Singh R. Zheng Y. Cao Z. Li M. (2018). GRF-interacting factor1 Regulates Shoot Architecture and Meristem Determinacy in Maize. *Plant Cell* 30 360–374. 10.1105/tpc.17.00791 29437990 PMC5868708

[B58] Zhang Z. Yan Y. Tian Y. Li J. He J.-S. Tang Z. (2015). Distribution and conservation of orchid species richness in China. *Biolog. Conserv.* 181 64–72. 10.1016/j.biocon.2014.10.026

