# Supplementary figures and images for: Organ-Specific Gene Expression Reveals the Role of the Cymbidium ensifolium-miR396/Growth-Regulating Factors Module in Flower Development of the Orchid Plant Cymbidium ensifolium
Source: Front Plant Sci. 2022 Jan 27;12:799778. doi: 10.3389/fpls.2021.799778 (PMC8829051; doi:10.3389/fpls.2021.799778)

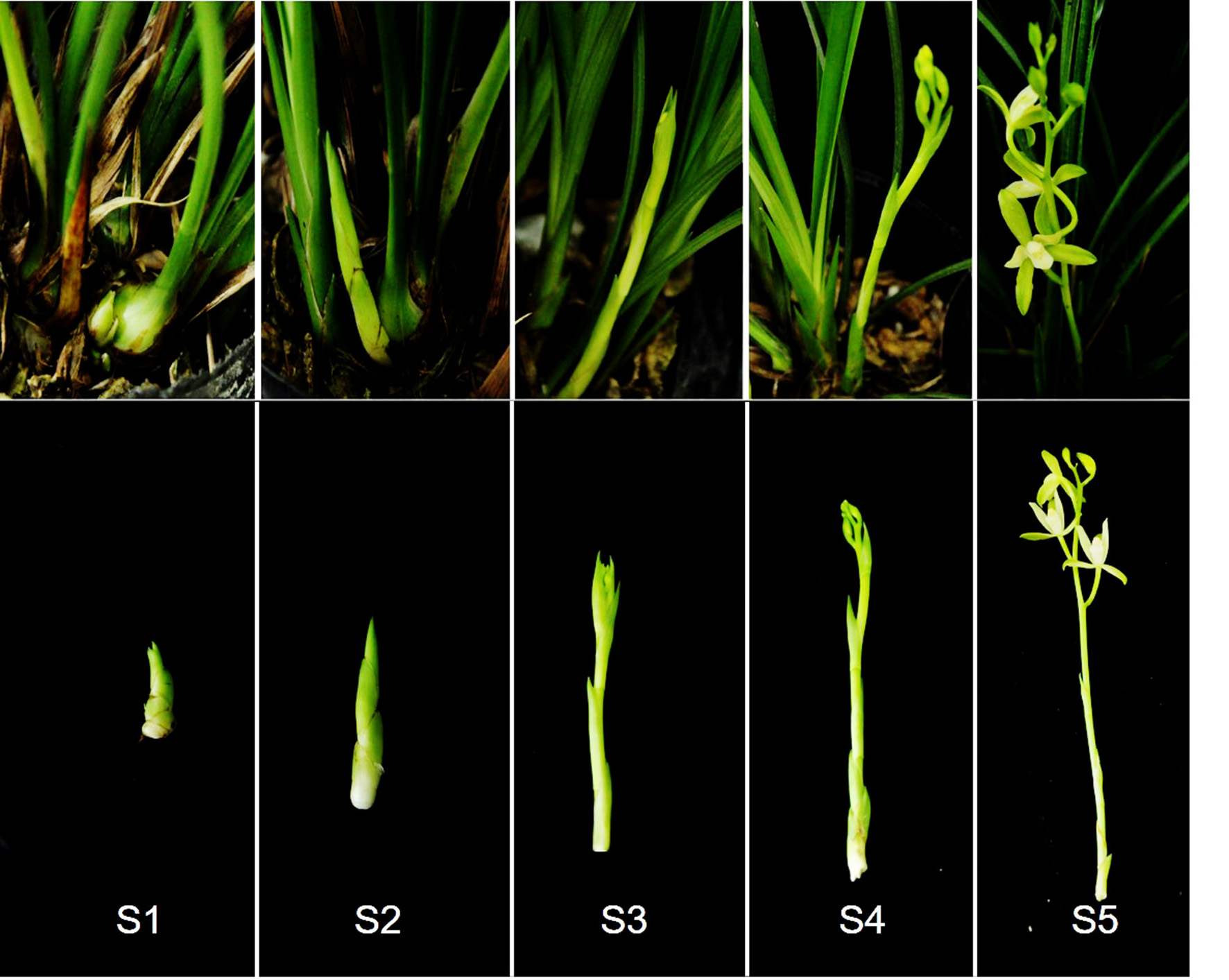

Supplement: Supplementary Figure 1 — Floral development process of Cymbidium ensifolium. [file Image_1.JPEG]

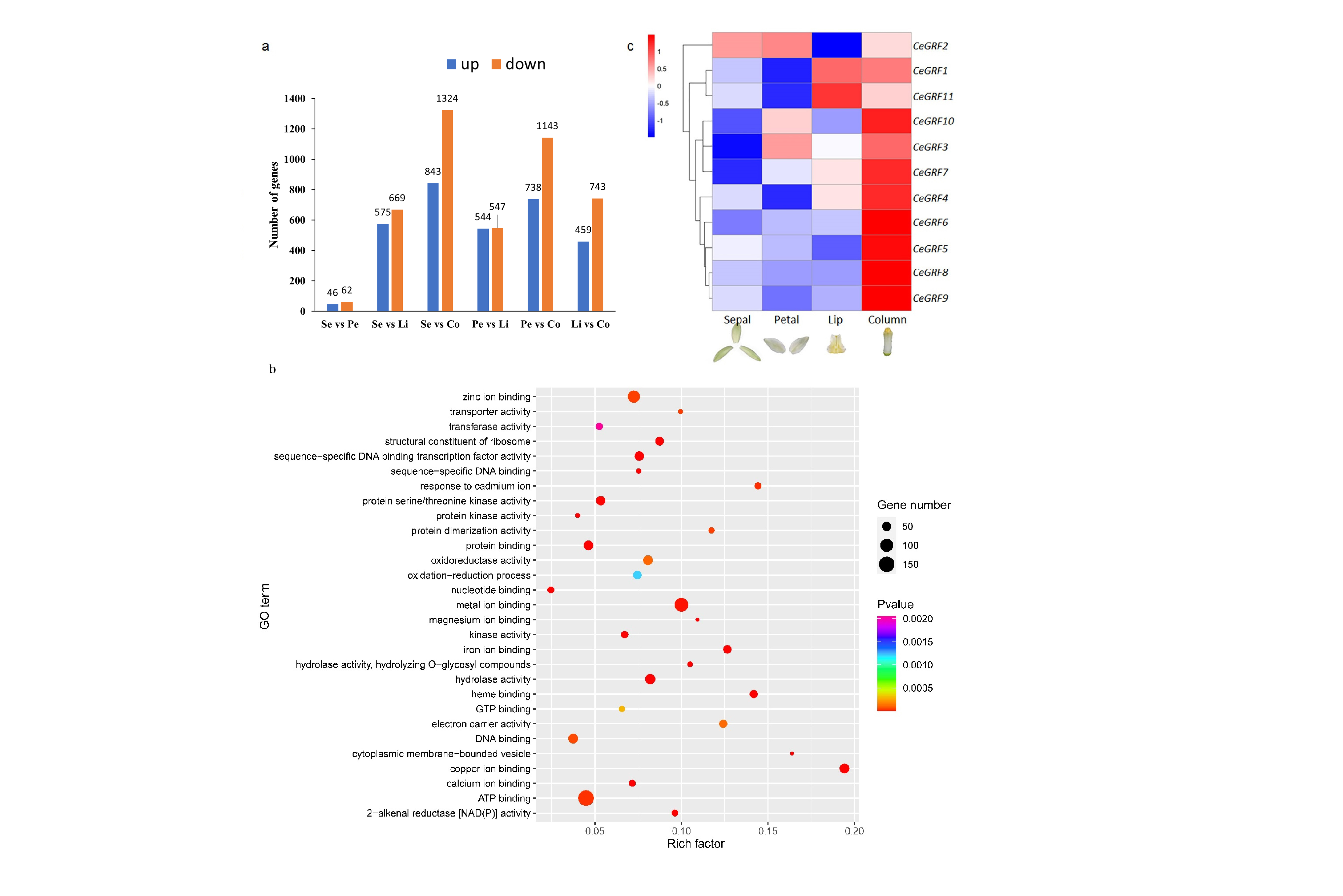

Supplement: Supplementary Figure 2 — Comparative transcriptome analysis of individual floral Organs. (A) Transcripts differentially expressed between different floral organs. Up- and down-regulated transcripts were quantified. The results of six comparisons between each two samples are shown. Se, sepal; Pe, petal; Li, Lip; Co, column. (B) GO term classification of differentially-expressed unigenes. (C) Expression heat map of CeGRF genes in sepal, petal, Lip, and column. Expression values from RNA-seq data were log2-transformed and are displayed as filled blocks in blue to red indicating gene expression intensity from low to high. [file Image_2.JPEG]

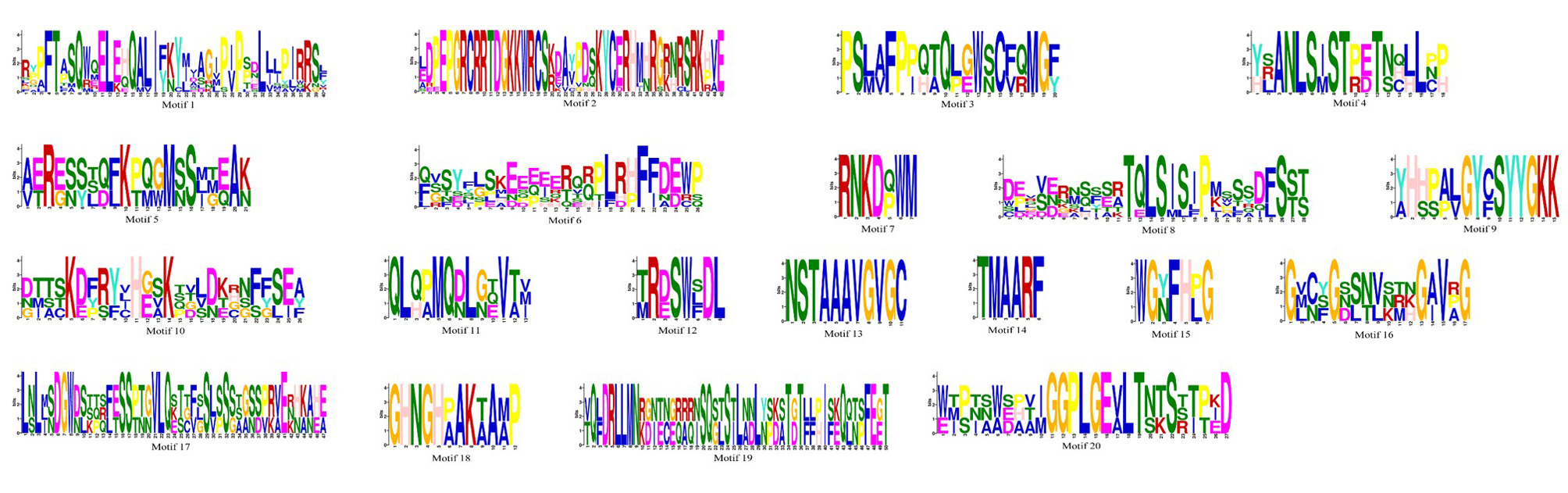

Supplement: Supplementary Figure 3 — Detailed information for protein motifs predicted in CeGRFs. [file Image_3.JPEG]

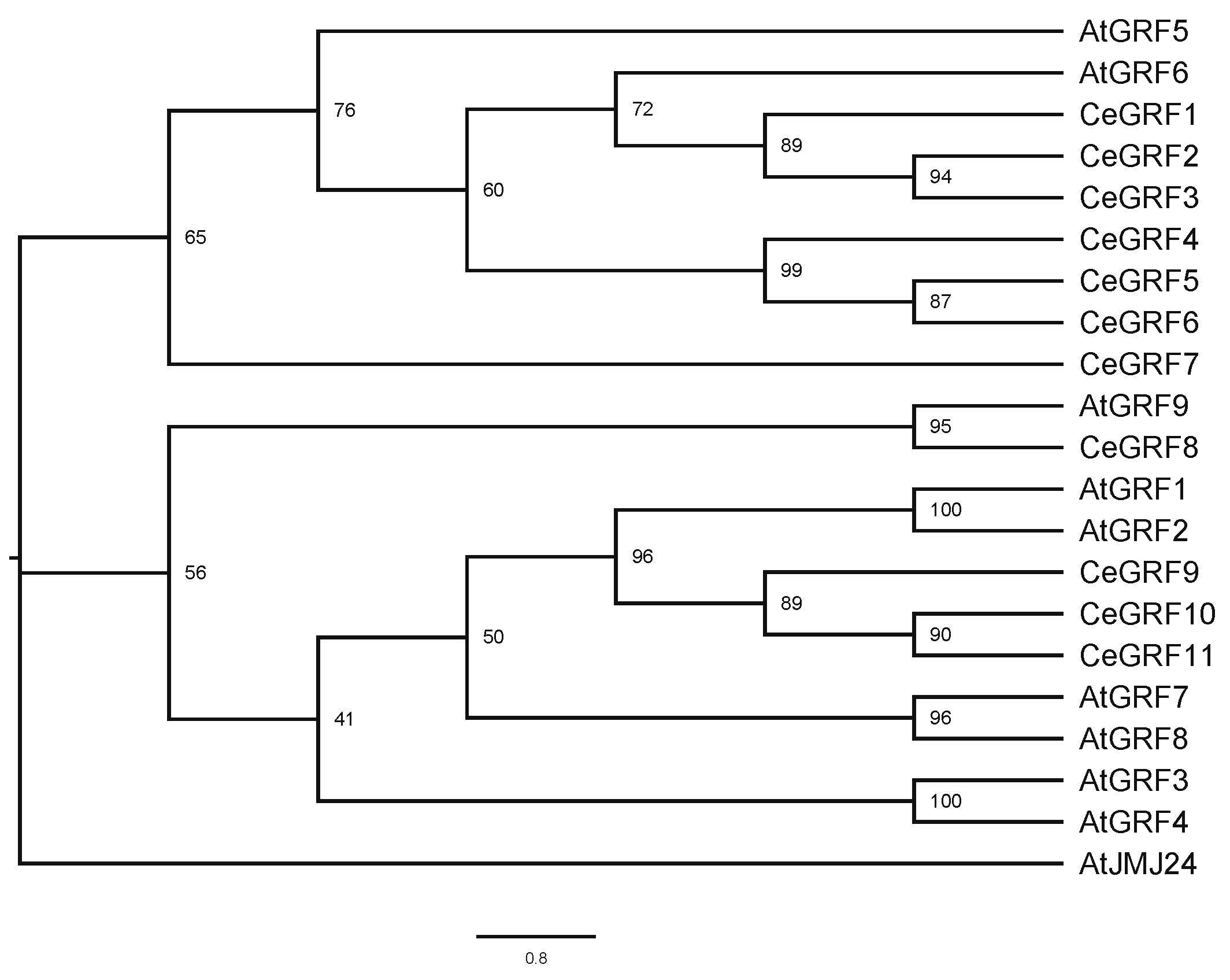

Supplement: Supplementary Figure 4 — Phylogenetic analysis of CeGRFs and AtGRFs. [file Image_4.JPEG]

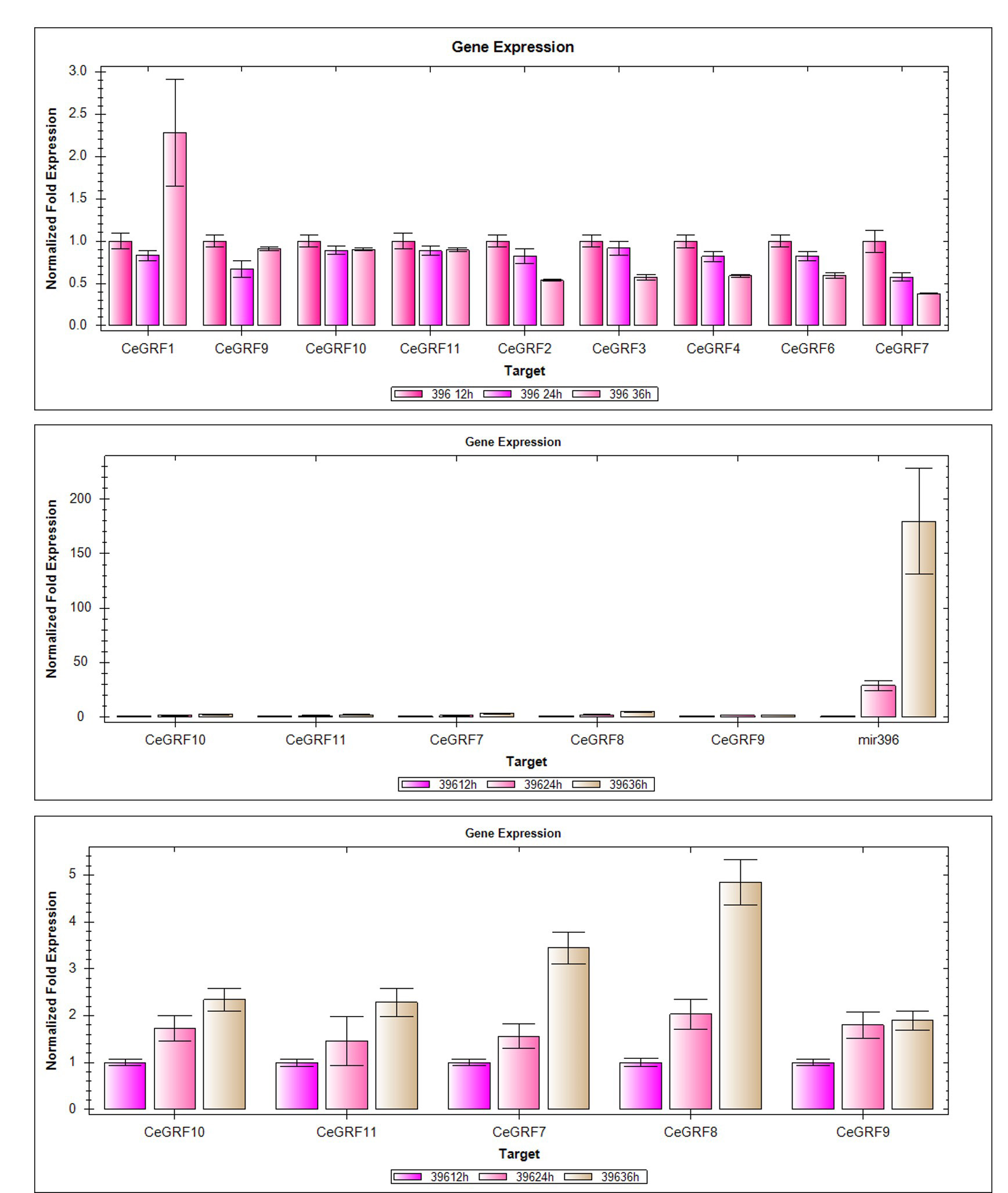

Supplement: Supplementary Figure 5 — Cymbidium ensifolium-miR396-regulated CeGRFs expression in a Cymbidium ensifolium protoplast-based transient expression system. [file Image_5.JPEG]

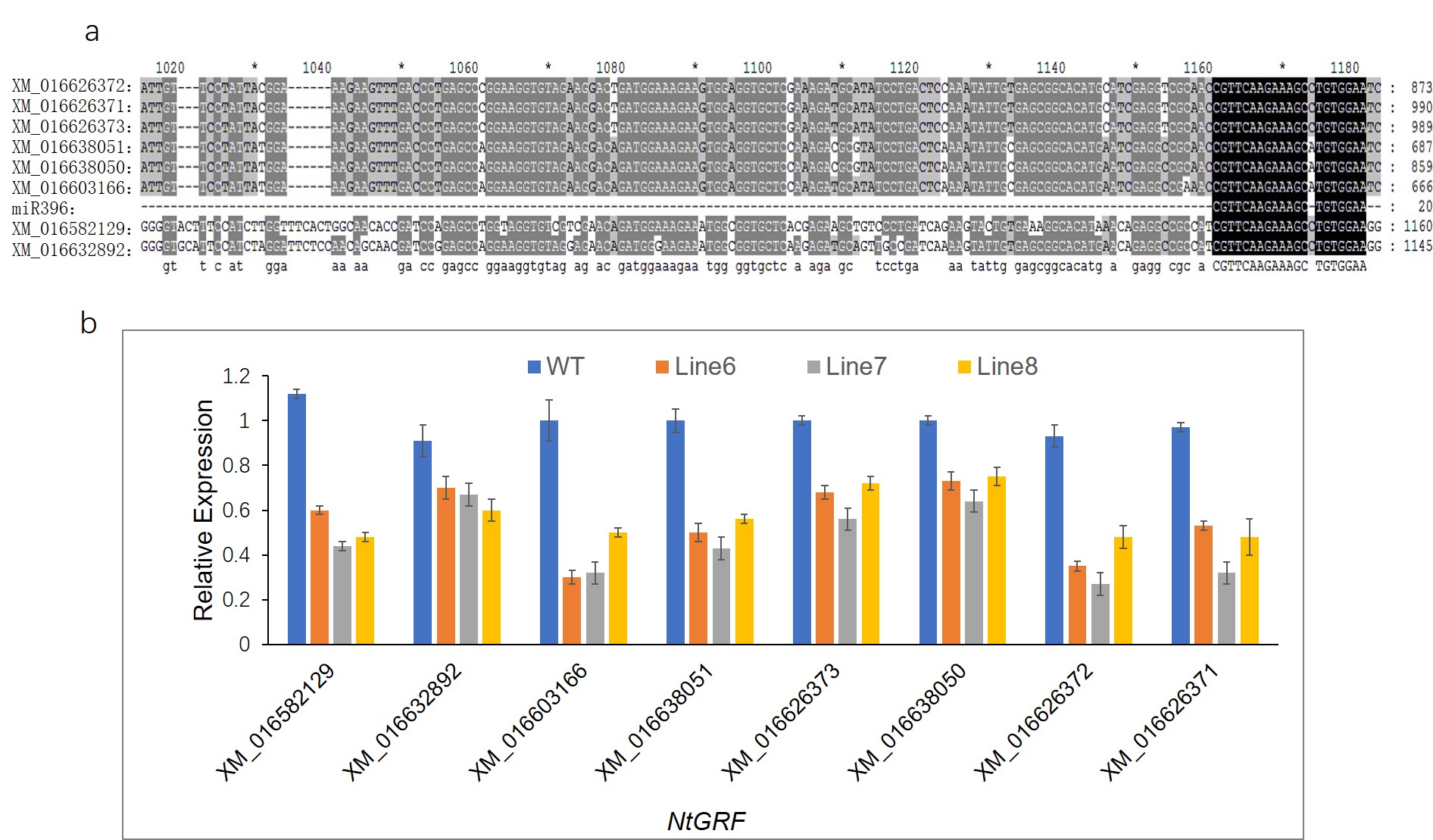

Supplement: Supplementary Figure 6 — Cymbidium ensifolium-miR396-regulated NtGRFs expression in transgenic tobacco plants. [file Image_6.JPEG]
