# Supplementary material for: Organ-Specific Gene Expression Reveals the Role of the Cymbidium ensifolium-miR396/Growth-Regulating Factors Module in Flower Development of the Orchid Plant Cymbidium ensifolium
Source: Front Plant Sci. 2022 Jan 27;12:799778. doi: 10.3389/fpls.2021.799778 (PMC8829051; doi:10.3389/fpls.2021.799778)
Supplement: Supplementary file 11 [file Table_5.DOCX]

Supplementary Table5. Sequence characteristics of CeGRF

| Serial No. | Gene Name | ORF(bp) | Protein | | | | |
| --- | --- | --- | --- | --- | --- | --- | --- |
|  |  |  | Length (aa) | Domain (Start-End) | | MW(kDa) | PI |
|  |  |  |  | QLQ | WRC |  |  |
| 1 | CeGRF1 | 921 | 306 | 14-22 | 91-94 | 35.7622 | 9.80 |
| 2 | CeGRF2 | 849 | 282 | 15-23 | 72-80 | 32.0888 | 8.41 |
| 3 | CeGRF3 | 780 | 259 | 14-22 | 71-79 | 29.8184 | 8.91 |
| 4 | CeGRF4 | 1110 | 369 | 34-42 | 86-94 | 40.9213 | 8.13 |
| 5 | CeGRF5 | 1017 | 338 | 18-26 | 65-73 | 37.4781 | 8.77 |
| 6 | CeGRF6 | 1110 | 369 | 18-26 | 65-73 | 40.3036 | 7.72 |
| 7 | CeGRF7 | 783 | 260 | 21-29 | 74-82 | 30.1089 | 8.80 |
| 8 | CeGRF8 | 567 | 188 | 66-74 | 110-118 | 20.6136 | 8.97 |
| 9 | CeGRF9 | 1530 | 509 | 63-71 | 118-126 | 55.5772 | 9.62 |
| 10 | CeGRF10 | 1380 | 459 | 33-41 | 90-98 | 49.8710 | 8.48 |
| 11 | CeGRF11 | 1671 | 556 | 144-152 | 200-208 | 61.5399 | 7.30 |
